# Breast Cancer Classification Using FCN and Beta Wavelet Autoencoder

**DOI:** 10.1155/2022/8044887

**Published:** 2022-06-23

**Authors:** Hussah Nasser AlEisa, Wajdi Touiti, Amel Ali ALHussan, Najib Ben Aoun, Ridha Ejbali, Mourad Zaied, Ayesha Saadia

**Affiliations:** ^1^Department of Computer Sciences, College of Computer and Information Sciences, Princess Nourah Bint Abdulrahman University, P.O. Box 84428, Riyadh 11671, Saudi Arabia; ^2^Research Team in Intelligent Machines, National School of Engineers of Gabes, B. P. W 6072, Gabes, Tunisia; ^3^College of Computer Science and Information Technology, Al Baha University, Al Baha, Saudi Arabia; ^4^REGIM-Lab, Research Groups in Intelligent Machines, National School of Engineers of Sfax (ENIS), University of Sfax, Sfax, Tunisia; ^5^Department of Computer Science, Faculty of Computing and Artificial Intelligence, Air University, PAF Complex, Islamabad, Pakistan

## Abstract

In this paper, a new classification approach of breast cancer based on Fully Convolutional Networks (FCNs) and Beta Wavelet Autoencoder (BWAE) is presented. FCN, as a powerful image segmentation model, is used to extract the relevant information from mammography images. It will identify the relevant zones to model while WAE is used to model the extracted information for these zones. In fact, WAE has proven its superiority to the majority of the features extraction approaches. The fusion of these two techniques have improved the feature extraction phase and this by keeping and modeling only the relevant and useful features for the identification and description of breast masses. The experimental results showed the effectiveness of our proposed method which has given very encouraging results in comparison with the states of the art approaches on the same mammographic image base. A precision rate of 94% for benign and 93% for malignant was achieved with a recall rate of 92% for benign and 95% for malignant. For the normal case, we were able to reach a rate of 100%.

## 1. Introduction

In the early stages of breast cancer, a tumor is too small to be felt and it may not cause any symptoms, but it could be seen by using different imaging techniques [1]. Various researchers have investigated the domain of breast cancer for early diagnosis. Machine learning (ML) algorithms are used in computer-aided diagnosis (CAD) systems to help in breast cancer detection and classification [2–5]. All machine learning algorithms are based on common steps, starting from the mammogram preprocessing followed by segmentation then feature extraction and selection, and finally applying the machine learning algorithm to classify the mass in the breast as either benign or malignant [6]. Deep learning uses efficient techniques that move from handcraft to automatic image segmentation, feature extraction, and feature selection to achieve more accurate detection and classification results [7]. Fully Convolutional Networks (FCNs) [8] are one of the most successful state-of-the-art deep learning methods that replaced all the fully connected layers with convolutional layers. The fundamental idea behind this method is to employ CNN as a feature extractor to generate high-level feature maps. These maps are then further up-sampled to provide pixel-by-pixel output. The method allows for end-to-end training of CNN for semantic segmentation with input images of any size. Skip connections [9] also allow data to flow directly from low-level feature maps to high-level feature maps without distortions, to improve localization accuracy and speed convergence. Many studies used FCN to segment breast cancer masses. In our proposed model, we combine FCN and Beta wavelet autoencoder BWAE to extract the most relevant features and reduce the dimension of the mammogram image through a set of layers (encoding), then reconstruct the image with the most significant features (decoding) to be classified correctly.

The idea is to model only the masses that are likely to be abnormal. FCN will be used to extract strange masses in the breast. Instead of modeling the total image, WAE will model just strange masses. In this case, we will only model the useful information.

Our paper is structured as follows: Section 1 presents the Literature Review. Section 2 demonstrates our proposed method for breast cancer detection and classification. Section 3 lists the experimental results, and Section 4 concludes the paper.

## 2. Literature Review

Over the past several years, deep learning techniques have achieved superior performance accuracy over classical machine learning methods on a wide variety of applications, especially in the medical field [10–12]. Convolutional Neural Networks (CNNs) [13] are the most common deep architecture used for breast cancer detection and classification. The latest studies of breast cancer detection and classification have achieved different performance and accuracy with different image preprocessing techniques [14, 15], CNN architectures [16], activation functions [17], and optimization algorithms [18, 19], and whether it applied as patches or images [20, 21]. Many research studies show that the CNN overcomes the limitation of classical machine learning methods and achieved better results in the detection and classification accuracies of breast cancer [22, 23]. Moreover, the depth and width of the deep network can help to improve the network's quality [24]. A comprehensive survey was conducted by Michael et. al. [25] for all Breast *Cancer* Segmentation Methods. In their research, they review the *deep learning segmentation methods* that are used to extract masses from mammogram images and highlight the most frequently used of them. The segmentation techniques play an essential role in the diagnosis, feature extraction, and classification accuracy of breast masses as benign and malignant. Different deep learning segmentation methods are used for breast cancer images such as FCN [8], U-Net [9, 26, 27], Segmentation Network (SegNet) [28], Full Resolution Convolutional Network (FrCN) [29], mask Region-Based Convolutional Neural Networks mask (RCNNs) [11, 30], Attention guided dense up-sampling network(Aunet) [31], Residual attention U-Net model (RUNet) [32], conditional Generative Adversarial Networks (cGANs) [33], Densely connected U-Net and attention gates (AGs) [34], and Conditional random field model (CRF) [35].

U-Net [9] is one of the most frequently deep learning segmentation techniques that is used with mammograms to detect breast cancer. It is encoder-decoder architectures that modify and extend FCN by using skip-connection operations to replicate the feature maps of its corresponding down-sampling process (i.e., pooling) and then combine them (called a concat layer) to include image pixel context information in the up-sampling convolution process. It is a fast and efficient segmentation method that demonstrates excellent results in breast cancer mass detection and classification. The U-Net has numerous advantages for segmentation tasks: first, it allows for the simultaneous use of global location and context. Second, it works well with a small number of training samples. Third, it produces the segmentation map using an end to end pipeline process that preserves the whole context of the input images, which is a significant advantage in mammogram classification.

A full resolution convolutional network (FrCN) model was proposed by Al-antari et. al. [29] to segment breast cancer masses that are detected by using You-Only-Look-Once (YOLO) model [36]. Their segmentation method preserves the details of tiny objects by removing max-pooling and subsampling layers in the encoder network. The decoder of FrCN replaces all three FC layers with three full convolutional layers. The accuracy of their segmentation model is 92.97%, and the overall accuracy of their CAD system using the CNN classifier is 95.64%.

Many researchers used U-Net architecture [32, 37] with or without filters to detect and classify mammogram images, and some of them modify U-Net architecture to enhance the classification accuracy of breast cancer. Abdelhafiz D. et al. [32] proposed a residual attention U-Net model (RUNet) that uses the U-Net architecture and replaced its regular convolution layers by residual blocks with the identity connections to connect the encoder and decoder paths at the same level. The new architecture is deeper to preserve information and enhance the segmentation performance of mammogram images. The last layer uses a ResNet classifier that achieved 98% classification accuracy.

Conditional Generative Adversarial Networks (cGANs) [33] is a deep segmentation method composed of encoder and decoder which make up the generator network. The encoder layers extract the features from the input images, such as texture, edge, and intensity, while decoder layers construct a binary mask based on the extracted features. The generator network generates a mask for mammograms masses that is fed to the classifier. Li S. et. al. [34] proposed a breast mass segmentation method that is composed of densely connected U-Net with attention gates (AGs). The encoder in the U-Net architecture is densely connected to CNN to work as a feature extractor of different size and shape of breast masses, and the decoder is integrated with AGs to enhance the segmentation of the U-Net model.

Deep neural networks integrate the probabilistic model called the Conditional Random Field (CRF) model [38] to be used for breast cancer mammogram images segmentation. CRF is used initially in deep neural networks as postprocessing of FCN which model the correlations among pixels for semantic image segmentation to achieve sharper boundaries.

Dhungel N. et. al. [35] proposed a deep learning model that integrates CNN, CRF, and Structured support vector machine SSVM to segment breast cancer masses in and their model shows significant results.

On the other hand, deep learning algorithms are complex architecture and require a large amount of data for training, so the researchers use different techniques to overcome these issues such as data augmentation and transfer learning. Also, modifying the architecture of the deep learning model to enhance the computation complexity and learning time such as combining deep learning and wavelet Network.

Combining deep neural networks with Beta wavelet networks and sparse coding are used for automatic breast cancer detection and classification. Ben Ali, R. et al. [39] proposed Deep Stacked Patched Auto-Encoders (DSPAEs) framework to detect and classify medical images. Their method is applied on mammogram images that are encoded and decoded by an Autoencoder to reduce the dimension of the input data through a set of layers (encoding), then reconstruct a new representation with more relevant features (decoding). The last layer of their model uses a linear classifier to classify breast images with 97.54% and 98.13% classification accuracy for MIAS and DDSM datasets, respectively.

Our work in this paper is encouraged by our previous work, where we proposed in Hassairi S. et. al. [40] a Deep Stacked Beta Wavelet Auto-Encoder (DSBWAE) to classify images and speech signals using the global score of each wavelet. The model constructs deep wavelet AEs layers with linear classifiers at the last layer. The classification results achieved outperform other classifiers that used the same datasets.

In Table 1, we demonstrate the results of different breast cancer segmentation and classification methods based on Deep Neural Networks.

## 3. Proposed Approach

**Figure 1:**
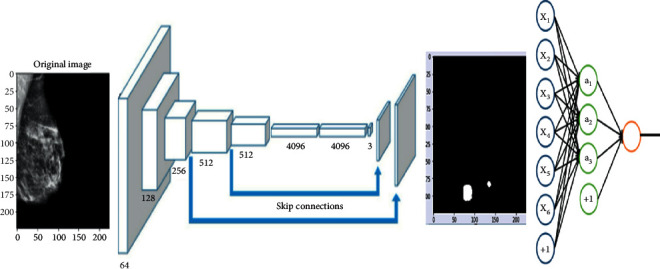
Illustration of the approach.

Figure 1 provides an overview of our proposed method for the detection and segmentation of a strange mass in the breast and the identification of the type of this mass.

To ensure proper segmentation and classification of masses in the breast, the proposed method makes use of two sequential modules. The segmentation approach is based on an FCN architecture. Features extraction approach is based on Beta wavelet autoencoder. The classification phase is based on a linear classifier.

Before proceeding with image segmentation, a preprocessing phase is required. The details of the preprocessing phase are detailed in the next section.

### 3.1. Preprocessing

The goal of image preprocessing is to get them ready as input for the model. The idea is to increase the quality of the images so that features can be retrieved more easily. An approach like this offers the model with more relevant features that it can learn efficiently. The following are the steps in the preprocessing pipeline:

- Cropping borders
- Normalization
- Removing the artifacts
- CLAHE enhancement

Raw mammography has the drawback of elucidating the procedures involved in the preprocessing of the chosen image.

The purpose of this pipeline is to fix these issues so that we can obtain high-quality mammography images for use by the model.

**Figure 2:**
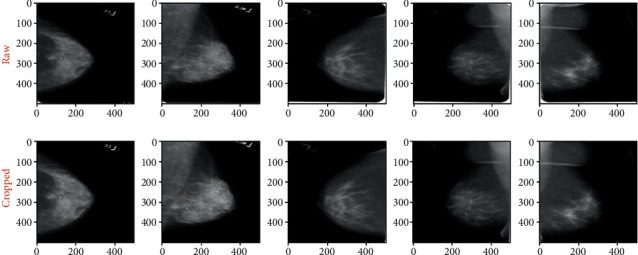
Original and cropped image.

**Figure 3:**
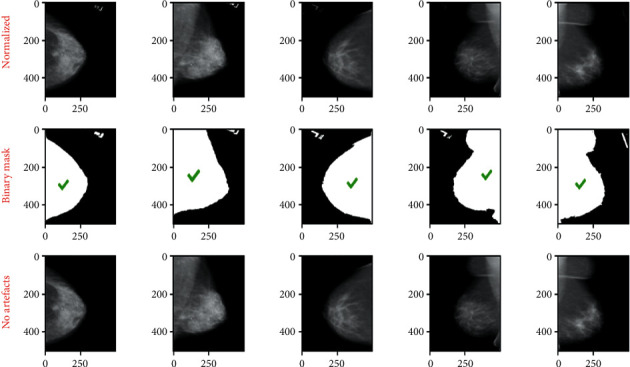
Images resulting from normalization and removing artifacts phases.

**Figure 4:**
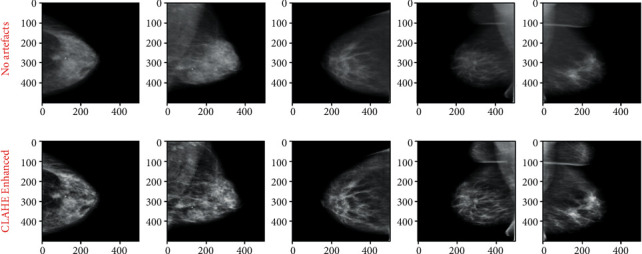
CLAHE enhancement

*Step 1*. Crop Borders: this step solves the problem of the bright white in borders and/or corners of raw mammograms. These white borders vary in thickness from image to image, which may cause huge issues in the model training phase, as they may consider these random edges as part of the features of mammograms. After a long process of trials and errors, we crop 1% of the image's width and 4% of the height.  Figure 2 shows the results of cropped images.
*Step 2*. Normalization: this step solves the problem of pixel value ranges from [0, 65535] to [0, 1]. The mammogram images are saved as 16 bit arrays. This means that the pixel values range from [0, 2^16^] these large values may slow down the learning process of the Neural Network. The Min-Max scaling consists in changing the range of pixel intensity values into a range that is more familiar or normal to the senses. Mathematically, the Min-Max Normalization equation is represented as follows:
  (1) $$\begin{array}{c} {\hat{M}}_{i,j}=\frac{M_{i,j}-\text{Min}\left(M\right)}{\text{Max}\left(M\right)-\text{Min}\left(M\right)}, \end{array}$$
where *M* is an image and *M*_i,j_ is a pixel.
*Step 3*. Artifacts Removal process:this step solves the problem of floating artifacts that appear in the background. In fact, these artifacts serve as markers that provide information like the orientation of the scan.  Figure 3 illustrates images resulting from the normalization and removing artifacts phases.
To remove the artifacts, we follow these steps. By visual inspection, we can say that most of the background pixels are very close to black (pixel values are close to 0). Therefore, we can binary the mammogram using a threshold value to create a binary mask, where 0 indicates background pixels and 1 indicates a pixel belonging to the breast or artifacts region. After generating a binary mask we expand the boundaries of the white region in the mask to ensure that we really capture the entire region of any artifacts. Now from another visual inspection, the breast contour is almost always the largest contour in the mask and always binaries as a single region. We can safely say that the largest contour in the mask is the only region we want to keep from the original mammogram.
*Step 4*. CLAHE enhancement:this step solves the problem of poor contrast of breast tissues. This causes the breast region to appear as almost monotonous gray, resulting in almost no textures and meaningful differences between breast tissues and mass abnormalities. Therefore, this may slow the model's learning. To solve this problem we apply contrast-limited adaptive histogram equalization (CLAHE) that adjusts the global contrast of an image by updating the image histogram's pixel intensity distribution; this helps enhance the small details, textures, and features from the mammogram.  Figure 4 shows the CLAHE enhancement of images.

### 3.2. FCN

**Figure 5:**
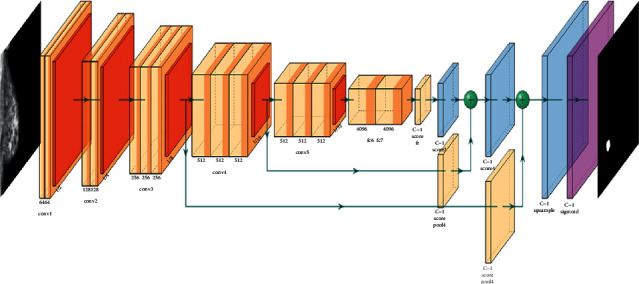
FCN8s model architecture.

A Fully Convolutional Network, or FCN, is an encoded and decoded architecture, primarily used for semantic segmentation of an image. The encoder part extracts the pixelwise feature maps from the input image while the decoder restores the original resolution without losing any information. FCNs can take advantage of well-known ConvNets models such as VGG and ResNet that have been previously trained for the classification task. In keeping with this idea, we took a VGG-16 (with 16 layers) model pretrained on the ImageNet dataset and transforms it to serve as an encoder. To accomplish this transformation first, we removed the fully connected layers and replaced them with 1 × 1 convolutional layers. This method speeds up the learning process and improves the efficiency of our model, by utilizing the knowledge stored in a pretrained VGG-16, this is known as the transfer learning technique. As for the decoder part that follows the encoder, we used another technique known as transposed convolutional layers to upsample the resulted pixelwise feature maps from the encoder. After preparing the encoder and decoder, skip connections are added, what this connection does is to connect the output of one layer to a nonadjacent layer. By doing so, the skip connection allows our model to use information from multiple resolutions. As shown in Figure 5 that resumes our model, the skip connections outputted from the third and fourth max-pooling layers to connect with the first and the second transposed convolutional layers respectively in decoder parts.

In a classic convolutional network with fully connected final layers, the size of the fully connected layers limits the size of the input. Passing images of different sizes will result in outputs of different sizes. In the last step, the matrix multiplication cannot be performed. On the other hand, convolutional operations basically do not care about the size of the input; a fully convolutional network will work on images of any size.

Fully convolutional networks have been able to solve the size problem in computer vision tasks. These architectures take advantage of the following three special techniques:

- Replace fully connected layers with one by one convolutional layers
- Up-sampling through the use of transposed convolutional layers
- Skip connections

**Figure 6:**
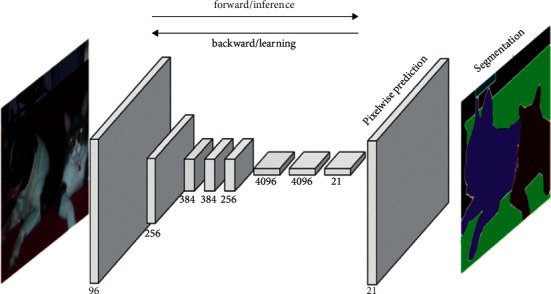
General architecture of an FCN [33].

This architecture, Figure 6, will use information from multiple resolution scales allowed from skipped connections. As a result, the network is able to make more precise segmentation decisions.

An FCN is composed of an encoder and a decoder. The encoder will extract relevant features that will be used by the decoder. FCN is a CNN with a 1^∗^1 convolutional layer as a fully connected layer. The encoder is followed by the decoder using transposed convolutional layers to up-sample the image. A skip connection is added between layers. A skip connection is a connection of a layer to a nonadjacent layer. This technique allows the network to use information from multiple resolutions. Figure 5 illustrates an example of FCN architecture.

Figure 5 presents a FCN8 model. This architecture is composed of the following parameters:

- Base model = VGG16-D
- Input shape = (224^∗^224^∗^3)
- Output shape = (224^∗^224^∗^1)
- Number of classes = 1
- Optimizer = Adam
- Learning rate = 1e-05
- Metrics = Accuracy and Intersection over Union (IoU)
- Loss function = BinaryCrossentropy
- Batch Size = 32
- Epochs = 50
- Steps_per_epoch = 506

**Figure 7:**
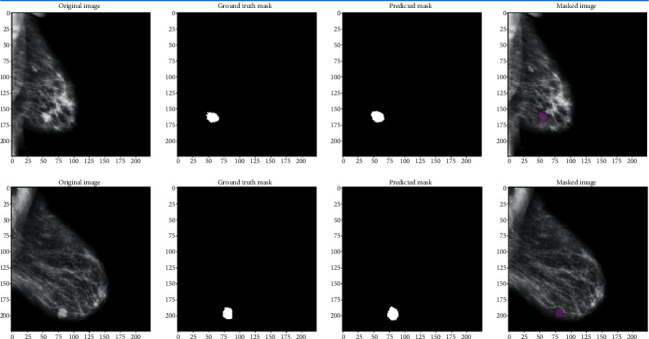
FCN8s segmentation results.

The segmentation phase is added to improve the modeling phase. Indeed, the images resulting from the segmentation phase have more useful information than the original images. Segmented images only present information describing the state of a cancerous nodule while a mammographic image may contain additional information unnecessary for the modeling phase and the classification phase.  Figure 7 illustrates the results of the segmentation phase based on FCN8.

### 3.3. Autoencoder-Based Beta Wavelets Network

Based on the capacity of wavelet analysis and the AE approaches in feature extraction and learning, Hassairi et al. in [40] have implemented a BWAE. This architecture is based on a wavelet network (WN) and autoencoder. These two models have led to the DSBWAE from a new model of WN called Global Wavelet Network GWN.

To construct the GWN, we created a WN using the Best Contribution algorithm (BCA) [40]. It is based on WN using 2D FWT and multiresolution analysis. Each WN model has a signal. All WN of a class will be merged to construct GWN.

The creation of a GWN to approximate only one class from the dataset is considered as the first step. To get this GWN, we need to choose the best wavelets from all wavelets that are used in the decomposition of all signals of the dataset. Therefore, it is essential to create a WN for each signal and then calculate wavelets' scores to get the GWN that approximates only one class from the dataset [40].

**Figure 8:**
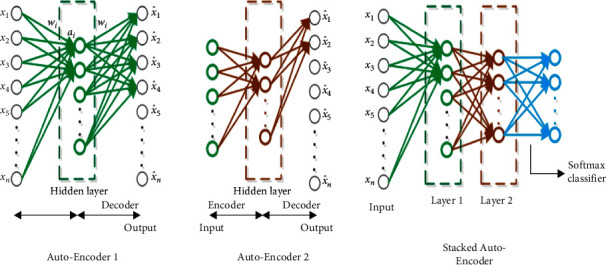
DSWAE with two layers.

To prepare a DSWAE, a set of WAEs is constructed. The association of WAEs led to a DSWAE. The hidden layer of the first WAE constitutes the input layer for the second WAE.  Figure 8 illustrates the construction of a DSWAE.

The linear function inside the neurons is transformed to a sigmoid function to allow the application of the fine-tuning. This function, the sigmoid function, favors the important features and derives the activation function in the back propagation step.

The Fine tuning [41] is commonly used in DL. Also, this step is used to greatly improve the performance of a stacked AE.

An intelligent pooling [42, 43] is used to optimize the quality of features in the hidden layers. When the wavelets found in the adjacent neurons have the same scale and the same type, we apply a pooling on these neurons.

**Figure 9:**
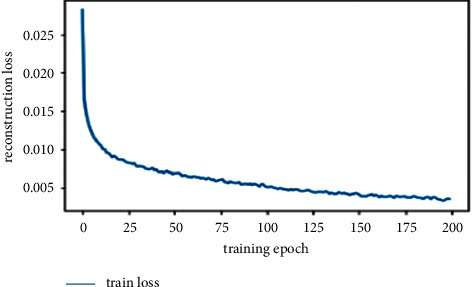
Learning curves of training the autoencoder model.

First, we trained the autoencoder in an unsupervised manner on normal and abnormal mammogram images, then we transferred the learning space from the autoencoder to the hybrid classification autoencoder, and then we trained the fully connected layer of the hybrid model using the fine-tuning technique.  Figure 9 illustrates the results of the training phase of the autoencoder.

**Figure 10:**
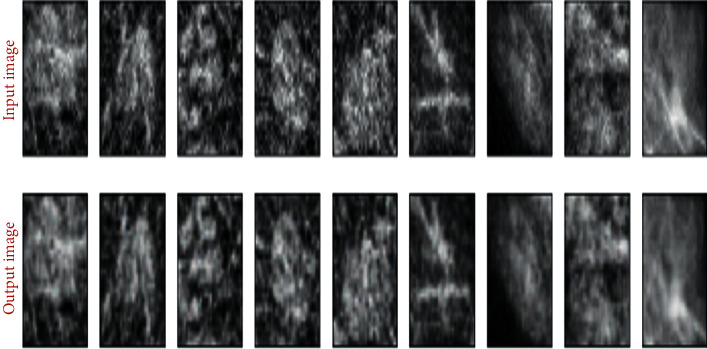
Autoencoder images reconstruction results.

Figure 10 illustrates the output of the autoencoder of training images. The output images illustrate the good quality of modeling due to the segmentation phase. This phase, based on FCN8, made it possible to keep only the useful part of the mammographic image.

## 4. Experimental Results

### 4.1. Dataset

To train and test our architecture, we used CBIS-DDSM presented in [44]. It is a database of mammographic images of breasts containing a mass. DDSM is composed of 2,620 scanned film mammography studies. It contains normal, benign, and malignant cases with verified pathology information. In our case, we were interested in the images where he has a mass to check if the mass is benign or malignant.

### 4.2. Dataset Splitting

For the segmentation model, we divided the dataset in two folders. One folder contains the full mammogram images; the other folder contains the ground truth masks. To train the FCN, we divided the dataset on 80% for the training phase and 20% for the testing phase. As a result, we get cropped images of the mass abnormality dataset for the classification phase. We used 80% of the new dataset for the training phase and 20% for the testing phase of the classification approach.

### 4.3. Classification

**Figure 11:**
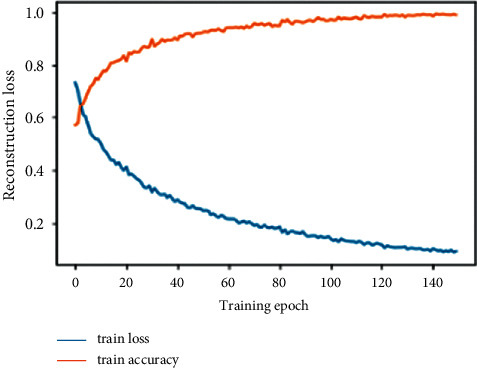
Learning curves of training hybrid classification autoencoder model.  Table 2 shows the confusion matrix presenting the classification rates resulting from our approach.

The classification process is based on a linear function. The SoftMax function was used at the end of the modeling process to identify the class of each image.  Figure 11 illustrates the learning rate of the segmentation and modeling phase.

### 4.4. Evaluation Metrics

The true positive is a consequence where the predictive model accurately predicts the positive class. While the true negative in contrast is a consequence where the predictive model accurately predicts the negative class. Whereas the false positive is a result where the predictive model wrongly predicts the positive class. While a false negative unlike a false positive is a result where the predictive model mistakenly predicts the negative class.

We compared our approach to several state-of-the-art approaches.  Table 3 presents an evaluation in terms of classification rates of the different approaches.

The evaluation details of our approach are shown in Table 4.

According to Tables 3 and 4, we can notice the effectiveness of our approach. This quality is due to the following two criteria:

- The feature extraction phase is based on the FCN. We no longer process the raw image. Based on the FCN, the part containing the useful information is segmented, which greatly reduces the modeling of the unnecessary information.
- The use of autoencoders based on wavelet networks. We know well the capacity of wavelet analysis, namely, the data modeling phase based on wavelet networks. The autoencoder based on wavelet networks is the association of a set of wavelet networks. So this autoencoder will have wavelet analysis capability and wavelets network modeling capability.

## 5. Conclusion

The intelligent analysis and classification of medical images has been a growing field in recent years. In this context, we have proposed a new vision for the classification of mammography images of breasts containing nodules. The proposed approach is a hybridization of two techniques. The FCN is used for nodule segmentation and localization of the affected part of the breast. This will remove unnecessary information from a mammogram image. In the end, we will be able to have just the image of the nodule from which we will decide on its quality. The DSWAE is an autoencoder model based on wavelet networks. This WEA brings together the qualities of wavelet analysis and the modeling qualities of wavelet networks. We have shown the performance of our approach compared to other state-of-the-art methods on the same test basis. These results justified our choice of techniques for the identification and classification of breast mammograms. The proposed method can also be useful for other types of cancers such as skin cancer [48], lung cancer [49], and brain tumor [50] detection.

## Figures and Tables

**Table 1:** List of studies used different deep segmentation methods of breast cancer mammograms images.

Ref#	Year	Segmentation method	Segmentation accuracy (dice coefficient index)	Classifier	Dataset	Classification accuracy
[37]	2020	Vanilla U-net	95.1%	VGG-16	CBIS-DDSM, INbreast, UCHCDM, BCDR-01	92.6%
[32]	2019	RU-Net	98.3%	ResNet	INbreast	98.7%
[34]	2019	U-Net integrated AGs	82.24%	—	DDSM	78.38%
[29]	2018	FrCN	92.69%	CNN	INbreast	95.64%
[35]	2015	CRF	90%	—	DDSM-BCRP and INbreast	—
[33]	2020	cGAN	98%	CNN based on BI-RADS	Abreast	97.85%
[39]	2020	DSPAE	—	Linear classifier	MIAS DDSM	97.54% 98.13%

**Table 2:** Confusion matrix.

		Benign	Malignant	Normal
Predicted	Benign	473	41	0
	Malignant	30	555	0
	Normal	0	0	500

**Table 3:** Classification rate evaluation.

	Global accuracy	Approach
Abdelhafiz et al. [37]	0.926	VGG-16
Tsochatzidis et al. [45]	0.81	Content-based image retrieval approach
Rouhi et al. [46]	0.79	Region growing and CNN segmentation
Xie et al. [47]	0.68	ELM
Our approach	0.95	FCN + WAE

**Table 4:** Classification metrics.

	Precision	Recall
Benign	0.94	0.92
Malignant	0.93	0.95
Normal	100	100

## Data Availability

To train and test our architecture, we used CBIS-DDSM presented in [36]. It is a database of mammographic images of breasts containing a mass. DDSM is composed of 2,620 scanned film mammography studies. It contains normal, benign, and malignant cases with verified pathology information. In our case, we were interested in the images where he has a mass to check if the mass is benign or malignant.
